# Beyond Iron: The Roles of CD71 in the Pathophysiology of Cancer—A Comprehensive Review

**DOI:** 10.3390/jcm14238265

**Published:** 2025-11-21

**Authors:** Georgios S. Markopoulos, Yannis V. Simos, Konstantinos I. Tsamis, Konstantina Gartzonika, Dimitrios Peschos, Lampros Lakkas

**Affiliations:** 1 Department of Physiology, Faculty of Medicine, School of Health Sciences, University of Ioannina, 45110 Ioannina, Greece; gmarkop@uoi.gr (G.S.M.); isimos@uoi.gr (Y.V.S.); ktsamis@uoi.gr (K.I.T.); dpeschos@uoi.gr (D.P.); 2 Department of Microbiology, Faculty of Medicine, School of Health Sciences, University of Ioannina, 45110 Ioannina, Greece; kgartzon@uoi.gr

**Keywords:** transferrin receptor 1 (CD71), iron metabolism, tumor microenvironment (TME), immune evasion, targeted cancer therapy

## Abstract

CD71, also known as transferrin receptor 1 (TfR1), is a transmembrane glycoprotein essential for cellular iron uptake, a prerequisite for cell proliferation, differentiation, and survival. While its canonical role in iron metabolism is well documented, accumulating research highlights CD71’s broader involvement in oncogenic processes that extend far beyond iron regulation. This review provides an overview of recent advances in understanding the multifaceted roles of CD71 within cancer biology. Specifically, we examine how CD71 participates in key cancer-related mechanisms, including modulation of intracellular signaling cascades, such as MAPK/ERK and PI3K/AKT pathways. We also mention the impact on regulation of apoptosis and autophagy, and orchestration of dynamic interactions within the tumor microenvironment such as adhesion/invasion and immune evasion. Emerging evidence indicates that CD71 not only sustains tumor growth and cancer cell proliferation through enhanced iron acquisition but also promotes metastasis, immune evasion, and therapy resistance. Moreover, CD71 contributes to the crosstalk between cancer and chronic diseases, with notable implications for cardiac, neurological, and gastrointestinal conditions often linked to malignancy. Through a comprehensive synthesis of current findings, we elucidate the complex and evolving roles of CD71 in cancer progression and therapy and provide an up-to-date account of its biology and pathophysiology. This review underscores the importance of understanding CD71 not only in oncology but also across the broader field of chronic diseases associated with malignancy. We suggest that future work should advance innovative therapeutic approaches that leverage CD71’s dual roles in cancer biology and systemic disease management to improve patient outcomes.

## 1. Introduction

Cancer remains one of the most challenging global health challenges, standing as the second leading cause of morbidity and mortality across diverse populations. Estimates show that cancer affects about one in five people over a lifetime, and causes death in nearly one in nine men and one in 12 women [1]. Forecasts project ~30.5 million cases and ~18.6 million deaths by 2050—driven largely by population growth and aging—while the target for decline in age-standardized mortality target require equity-focused cancer-control efforts [2]. Despite substantial advances in cancer research, prevention, and treatment, many types of malignancies continue to evade current therapeutic strategies, underscoring the urgent need to identify molecular mechanisms associated with tumor progression that may ultimately enable the identification of novel therapeutic targets. Among several molecules implicated in cancer biology, CD71, also referred to as transferrin receptor 1 (TfR1), has emerged as a protein of considerable interest [3].

CD71 is a homodimeric transmembrane glycoprotein that enables receptor-mediated iron uptake, a process essential for cell development and proliferation [4]. The metabolic demands of rapidly dividing cells, cancer cells in particular, may exhibit an increased dependency on iron, frequently accompanied by the overexpression of CD71 [3]. Importantly, Lyons et al., using the human transferrin receptor (CD71) as an affinity ligand, could capture diverse cancer cells from blood without prior knowledge of tumor type; capture efficiency and purity depend on CD71 expression, which varies with the cell cycle. Under optimized conditions, CD71-based capture achieved >80% purity, supporting CD71 as a promising target for liquid biopsy applications [3]. The phenomenon of iron dependency, also known as “iron addiction”, is a hallmark of many malignancies and reflects the central role of CD71 in supporting tumor growth and survival [5], including a role in ovarian cancer [6].

While CD71’s role in iron uptake is well recognized, its broader impact on cancer cell behavior and the tumor microenvironment remains less defined. Traditionally, CD71 has been studied almost exclusively within the context of iron metabolism and receptor-associated iron uptake [7]. However, emerging evidence has significantly expanded our understanding of this receptor, revealing that its role extends well beyond its canonical function in iron homeostasis and provides a link to essential cellular functions. CD71 is now recognized as a multifunctional molecule implicated in a diverse array of cellular processes in cellular physiology or pathology. For instance, Kawabata highlights that transferrin-bound iron is taken up primarily through CD71/TfR1 via clathrin-dependent, receptor-mediated endocytosis, while also noting CD71’s ability to internalize ferritin and serve as an entry receptor for certain pathogens [8]. CD71 plays also important roles in haematopoiesis, highlighting cell-intrinsic and possibly lineage-specific roles in proliferation and differentiation, as well as in functional polarization [9,10,11]. Significantly, TFRC (gene encoding CD71) loss-of-function causes an inborn error of immunity with impaired lymphocyte activation/proliferation due to defective iron uptake, providing evidence on an iron-immunity axis [12].

Emerging research indicates that CD71-associated processes are also involved in the pathophysiology of chronic diseases that frequently coexist with or predispose individuals to cancer. For example, dysregulated iron metabolism and/or CD71 expression has been implicated in the development of cardiac disorders, such as heart failure and arrhythmias, as well as in neurodegenerative diseases, including Alzheimer’s disease and Parkinson’s disease [13]. CD71 expression has also been negatively associated with selenoprotein P concentration in hospitalized heart failure patients [14]. Human failing myocardium in chronic heart failure patients shows altered expression of iron-metabolism proteins (including reduced expression of CD71), aligning cardiac dysfunction with disturbed iron handling [15]. In in vitro and in vivo preclinical models, knocking down transferrin receptor reduced atrial fibrillation by limiting ferroptosis and atrial fibrosis, directly linking CD71/iron pathways to arrhythmia [16]. Reviews synthesize that brain iron dysregulation and altered expression of iron transporters (including transferrin receptors) contribute to Alzheimer’s Disease (AD) pathology and glial dysfunction. Therapeutic antibodies that use CD71 as a blood–brain barrier (BBB) shuttle enhance microglial metabolism and target AD pathology in models, highlighting disease-relevant CD71-associated processes [17]. In Parkinson’s Disease (PD), the brain exhibits iron accumulation and redox imbalance (although variable), with emerging work proposing iron dysregulation as an additional feature in PD, while targeting iron pathways might be an attractive therapeutic strategy [18]. This intricate relationship between cancer and chronic diseases highlights that there may be systemic implications of CD71 dysfunction, suggesting that it might serve as a key molecule connecting tumor biology with chronic disease mechanisms. Since iron metabolism is deeply intertwined with tumor biology (proliferation, oxidative stress, immune regulation), recent evidence supports CD71 as a node connecting cancer to whole-body iron homeostasis [19].

While CD71’s role in iron uptake is well recognized, its broader impact on cancer cell behavior and the tumor microenvironment remains less defined. The purpose of this review is to synthesize canonical and non-canonical functions and outline translational opportunities of CD71 in cancer and chronic disease. Specifically, we aim to explore the mechanisms by which CD71 contributes to tumor growth, immune evasion, and therapy resistance, as well as its involvement in modulating the tumor microenvironment. Additionally, we will discuss the emerging evidence linking CD71-mediated iron dysregulation to chronic diseases, with a particular focus on cardiac and neuronal disorders. By synthesizing the latest research, this review seeks to illuminate the expanding role of CD71 in oncology and its potential to bridge the gap between cancer therapy and the management of associated chronic diseases. Ultimately, we aim to underscore the significance of CD71 in cancer biology, highlighting possible therapeutic interventions and clinical approaches to cancer treatment and patient care.

## 2. CD71 and Iron Metabolism in Cancer

Iron metabolism plays a fundamental role in cellular proliferation, differentiation, and survival, making it an indispensable process in both normal physiology and pathological conditions such as cancer. Iron is an essential trace element for cell growth and basic functions, yet excess iron can be harmful and carcinogenic. Iron overload drives reactive oxygen species, lipid peroxidation, and DNA/protein damage, promoting carcinogenesis or triggering ferroptosis. Iron also shapes the tumor microenvironment, metastasis, genomic stability, and epigenetic control, and cancer cells rewire iron acquisition, storage, and export [20], with CD71 playing significant roles in this process [21].

### 2.1. CD71-Mediated Iron Uptake

CD71 binds transferrin, a plasma glycoprotein responsible for transporting iron throughout the bloodstream, and facilitates its internalization via receptor-mediated endocytosis. Inside the cell, transferrin-bound iron is released in the acidic environment of the endosome and subsequently transported into the cytosol, where it is utilized in a variety of biochemical processes, including heme synthesis, electron transport, and the enzymatic activity of ribonucleotide reductase, a key player in DNA synthesis [8,22,23,24,25].

Cancer cells regularly exhibit unique metabolic reprogramming, which supports rapid proliferation and survival under harsh environmental conditions [26], while these metabolic changes are oncogene-driven, not mere by-products of damaged mitochondria [27]. Among these metabolic adaptations, there is a heightened need for iron to sustain the increased rates of DNA replication, mitochondrial respiration, and biosynthetic processes characteristic of tumor cells [28]. Contemporary summaries reaffirm that tumors display a higher dependency on iron than non-malignant tissues, linking this to growth and therapy vulnerabilities [29]. Consequently, cancer cells frequently overexpress CD71 to meet this demand, and its upregulation has been documented across a broad spectrum of tumor types [30], including breast [31], lung [32], hepatocellular [33], prostate cancer [34], among others. Elevated CD71 expression is strongly associated with aggressive tumor phenotypes, enhanced metastatic potential, and poor patient prognosis, highlighting its critical role in cancer progression. For example, in breast cancer, high CD71 correlates with poor outcome and endocrine resistance, while in hepatocellular carcinoma, CD71 overexpression predicts recurrence and worse survival [31,33]. In addition, gastrointestinal tumors show worse prognosis when expressing high CD71 [35] and lung cancer studies identify that blocking CD71 inhibits the growth of cancer cells [32].

In conclusion, CD71 as transferrin–iron entry in proliferating cells, links clathrin-mediated endocytosis to endosomal iron release and cytosolic use. In cancer, this flux sustains DNA synthesis, mitochondrial respiration, and redox programs that enable rapid growth. Because uptake is rapid and constitutive, CD71 represents a metabolic dependency and a drug-delivery entry point.

### 2.2. Transcriptional Regulation of TFRC Gene

**Figure 1:**
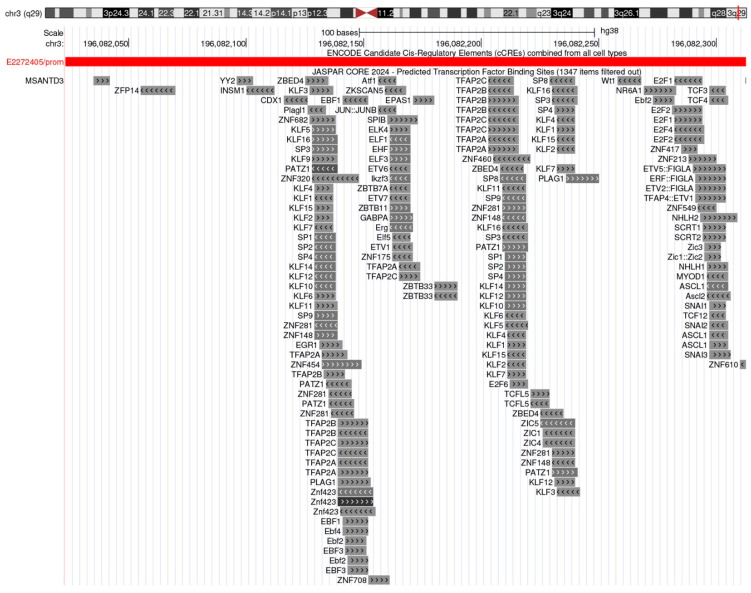
**Predicted transcription factor binding landscape at the human TFRC (CD71) proximal promoter.** UCSC Genome Browser snapshot (hg38; chr3:196,082,050–196,082,350; scale = 100 bp) showing ENCODE Candidate Cis-Regulatory Elements (cCREs; combined across cell types) and JASPAR CORE 2024 Predicted Transcription Factor Binding Sites. A promoter-class cCRE (ID E2272405/prom) marks the TFRC promoter region. The JASPAR track highlights dense clusters of GC-rich SP/KLF motifs (SP1-4; KLF1-16) alongside predicted sites for TFAP2 family (TFAP2A/B/C), E2F factors (E2F1-6), and ETS/related factors (ELK4, ELF1/3), among others (e.g., ZBTB7A, PATZ1). This architecture is characteristic of housekeeping, GC-rich promoters and aligns with TFRC’s proliferation-linked regulation. Data source: UCSC Genome Browser [47]; tracks: ENCODE cCREs [48] and JASPAR CORE 2024 TFBS [49] (filtered for minimum score 400). Abbreviations: cCRE, candidate cis-regulatory element; TFBS, transcription factor binding site.

Recent research has illuminated molecular mechanisms underlying the overexpression of CD71 in cancer [30]. A key driver is the activation of hypoxia-inducible factors (HIFs), a family of transcription factors that are stabilized under hypoxic conditions commonly encountered in the tumor microenvironment [36]. HIFs directly upregulate CD71 expression to increase iron uptake, thereby supporting cancer cells survival in low-oxygen environments. Multiple studies mapped functional HIF-1 binding sites (HREs) in the TFRC gene and demonstrated HIF-1–dependent transcriptional activation and hypoxic induction of transferrin receptor [37,38,39]. Additionally, oncogenic signaling pathways such as those mediated by c-Myc, Ras, and PI3K/AKT have been shown to enhance CD71 transcription and promote iron accumulation in cancer cells. ChIP and functional assays show that c-Myc binds a conserved E-box in TFRC and drives its expression and tumorigenesis [40]. Oncogenic RAS transformation increases cellular iron, linked to TFRC upregulation and ferroptosis sensitivity [41]. PI3K/AKT pathway activation upregulates TFRC and increases iron influx [42]. These pathways act synergistically with HIFs, amplifying CD71 expression and reinforcing the tumor’s “iron-addicted” phenotype [43,44,45,46]. An overview of TF-binding to CD71 promoter is provided in Figure 1.

Overall, Hypoxia (HIF-1/2) and oncogenic drivers (MYC, RAS, PI3K/AKT) converge on the TFRC locus to enhance transcription and link iron demand in stress-adapted tumors. These inputs cooperate with iron-sensing post-transcriptional control (IRP/IRE) to amplify CD71 at the cell surface. This convergence explains CD71’s association with aggressive “iron-addicted” phenotypes and offers opportunities for therapeutic intervention.

### 2.3. CD71 Expression Across Tumors and Its Impact

Tumor iron handling reshapes the tumor microenvironment and cell signaling and affects proliferation, invasion, and therapy response [50,51]. Excess intracellular iron contributes to the formation of reactive oxygen species (ROS) via the Fenton reaction, a chemical process that generates highly reactive hydroxyl radicals [52]. While excessive ROS levels can be cytotoxic, cancer cells exploit moderate ROS production to drive pro-tumorigenic processes [53,54]. Specifically, ROS-induced oxidative stress promotes DNA damage, genomic instability, and the activation of survival pathways such as those of MAPK and NF-κB, which may further enhance tumor growth and resistance to therapy [55,56]. Additionally, iron overload has been implicated in the regulation of key cellular processes such as angiogenesis, epithelial–mesenchymal transition (EMT), and immune evasion. Iron status (deficiency or overload) modulates angiogenic programs via HIF/VEGF and endothelial ROS, indicating a bidirectional regulatory role in tumor angiogenesis [57,58]. In addition, recent work links iron/ferroptosis pathways with EMT plasticity and drug resistance, and ties tumor CD71 signaling to immunosuppressive macrophage polarization and broader immune evasion [59,60], underscoring its multifaceted role in cancer biology.

The TME is often characterized by iron dysregulation, with cancer cells that antagonize and taking iron from surrounding stromal and immune cells. Experimental work shows that tumor cells impose iron restriction on the microenvironment, reshaping macrophage function and broader immunity [60,61]. This creates a competitive advantage for tumor cells while impairing the anti-tumor activity of immune cells such as macrophages and T-cells, which rely on iron for their function [11,60,62,63,64]. Thus, CD71-mediated iron uptake may sustain the metabolic demands of cancer cells but may also contribute to the establishment of an immunosuppressive and pro-tumorigenic microenvironment [65,66,67].

The overexpression of CD71 in cancer has also sparked interest in its potential as a therapeutic target. Multiple reviews and preclinical programs identify CD71 as an attractive, widely overexpressed tumor antigen under active therapeutic investigation (e.g., antibodies or conjugates to anticancer agents) [68,69]. Strategies aimed at inhibiting CD71-mediated iron uptake include the use of transferrin-conjugated cytotoxic agents, monoclonal antibodies targeting CD71, and iron chelators designed to deprive cancer cells of iron. Among them, Transferrin–drug conjugates (e.g., Tf–doxorubicin) may selectively kill cancer cells and overcome multi drug resistance in leukemia [70] and breast cancer [71], anti-CD71 antibodies may act therapeutically [68] and iron chelators (such as deferasirox, triapine and others) have been shown to exhibit anticancer activity in preclinical/clinical settings [72,73]. Furthermore, emerging technologies such as nanoparticle-based drug delivery systems have been developed to exploit CD71’s high expression in cancer cells, offering selective delivery of chemotherapeutic agents directly to tumors. In this conceptual framework, TfR-targeted nanoparticles and liposomes enhance tumor-selective uptake via CD71, improving intracellular drug delivery [74,75,76]. Additionally, peptide-doxorubicin constructs that bind CD71 demonstrate targeted delivery to CD71-overexpressing tumors [77]. Probody–drug conjugate CX-2029 (anti-CD71) shows antitumor activity with a design to limit normal-tissue binding, illustrating clinical translation of CD71-targeting [78].

In summary, current evidence supports that CD71 plays critical roles in cancer biology, bridging metabolic requirements of tumor cells with an ability to adapt and thrive in their environment. CD71 is upregulated across many solid and hematologic cancers, with the highest levels in aggressive, highly proliferative subsets. Across cohorts, higher CD71 generally is linked to adverse clinicopathologic features and worse survival. Understanding the multifaceted contributions of CD71 to iron metabolism and cancer pathophysiology opens paths for development of targeted therapies and a potential for improved patient outcomes.

## 3. Non-Canonical Functions of CD71

While CD71’s canonical role in iron uptake and metabolism is well established, a growing body of evidence reveals that it serves additional, non-canonical functions that significantly contribute to cancer progression. In this section we cover four axes: (i) signaling (MAPK/ERK; PI3K/AKT), (ii) apoptosis and autophagy, (iii) adhesion/EMT/migration, and (iv) immune modulation within the TME. These roles illustrate CD71’s multifaceted nature in oncogenic processes. For each, we summarize core mechanisms and note implications for therapy and biomarker development.

### 3.1. Signal Transduction Pathways

CD71 is increasingly recognized as more than an iron transporter, with mounting evidence that it can modulate signaling programs governing proliferation, survival, and differentiation. A noncanonical role for CD71 was shown in intestinal epithelium independent of iron uptake, establishing signaling/functional effects beyond transport [79]. In cancer models, CD71 abundance has been linked to activity in the MAPK/ERK and PI3K/AKT cascades—two core oncogenic pathways—through mechanisms that appear at least partly independent of iron uptake. For example, perturbing CD71 levels or engagement can coincide with ERK phosphorylation and pro-proliferative signaling, and PI3K/AKT activation has similarly been observed in contexts of heightened CD71 activity, though the precise coupling steps remain to be clarified [80]. In addition, CD71 knockdown curtailed rapid ERK activation and proliferation programs, indicating a signaling role separable from mere iron import [80]. While ligand-independent clustering is a recognized way to trigger ERK for several receptors, direct CD71-specific evidence is limited. However, CD71 overexpression/cross-manipulation correlates with ERK activation in tumors [81]. Multiple studies report that TFRC knockdown or modulation suppresses PI3K/AKT (±mTOR) signaling in cancer, while CD71 engagement can also elicit AKT activation [82]. Current hypotheses on the above interaction might point to roles for membrane organization and interactions of the CD71 cytoplasmic tail with adaptor/kinase machinery. Delineating these connections could reveal therapeutic entry points in tumors where CD71 overexpression helps drive disease progression [68,83,84].

Viewing glioma as a regulatory network rather than a single-pathway disease, in a Vartholomatos et al. publication, CD71/TFRC emerges as a hub where hypoxia, inflammation, and therapy signals converge [85]. In gliomas, HIF-1 and NF-κB programs coordinate, among others, stress-response, survival and immune-evasion modules. CD71 takes part in this circuitry because HIF-1 and NF-κB crosstalk can drive TFRC transcription under hypoxia/inflammation, wiring iron import directly into the core network that sustains tumor growth and adaptation [39]. Elevated CD71 in glioma/GBM then fuels an iron-dependent subnetwork (proliferation, metabolic plasticity, redox signaling), which in turn feeds back on HIF/NF-κB nodes via ROS and cytokine loops—creating self-reinforcing motifs that might support aggressiveness and treatment resistance [86,87,88]. Deregulation of CD71 expression might, in part, explain the action of natural anti-glioma agents [89,90].

Collectively, CD71 abundance and engagement can interface with MAPK/ERK and PI3K/AKT signaling beyond iron transport, aligning with pro-proliferative and pro-survival states. Therapeutically, this supports evaluating CD71 both as a targeted delivery entry point and as a signaling-linked biomarker to guide combinations with MAPK/PI3K pathway inhibitors.

### 3.2. Regulation of Apoptosis and Autophagy

Beyond signal transduction, CD71 has been implicated in regulating apoptosis and autophagy, two critical pathways in cancer. For example, Src-mediated phosphorylation of CD71 enhances anti-apoptotic signaling and cancer cell survival [91]. Recentc work shows CD71 controls autophagosome biogenesis/closure and its depletion impairs autophagic flux, overexpression stimulates it independent of iron [92]. Because evasion of apoptosis is a cancer hallmark, high CD71 often tracks with pro-survival signaling and poorer outcomes, and experimental manipulation of CD71 or its ligands can shift apoptotic regulators (e.g., BCL-2 family) and caspase activity [93,94]. This anti-apoptotic tilt contributes to therapy tolerance, with reports linking CD71 to drug resistance phenotypes [95]. CD71 also interfaces with autophagy—now recognized to aid tumor survival under hypoxia and nutrient stress—and recent work shows CD71 can modulate autophagic flux in an iron-independent manner [96,97]. Significantly, the autophagic turnover of iron and ferritin, molecules, denoted as feritinophagy, is critical for iron homeostasis [98]. Because autophagy can either protect or kill cancer cells depending on the specific cellular context, CD71’s positioning on this axis suggests it may help set the apoptosis–autophagy balance that determines survival versus death in hostile tumor microenvironments [98,99,100].

The above data support that perturbation of CD71 shifts apoptotic regulators and modulates autophagic flux, influencing stress tolerance and treatment response. CD71-directed approaches to perturb the apoptosis–autophagy balance may potentially enhance cytotoxic or stress-inducing therapies.

### 3.3. Cell Adhesion and Migration

CD71’s contribution to cancer progression also encompasses cell adhesion and migration. By intersecting with adhesion/signaling machinery and endocytic trafficking that regulates integrins, CD71 can influence cytoskeletal organization, thereby enhancing motility and invasiveness. Reviews of CD71 in cancer summarize evidence that TFRC elevation is associated with increased migration and invasion across tumor types [30], while TFRC knockdown reduces migration/invasion in osteosarcoma cells, supporting a pro-motility role [101]. While direct CD71–integrin binding in cancer remains under study, integrin trafficking/signaling is tightly coupled to clathrin/AP2 pathways that also handle CD71, providing a mechanistic intersection that can influence adhesion and the cytoskeleton [102].

A central program in metastasis is epithelial–mesenchymal transition (EMT) [103]. CD71 overexpression has been linked to EMT-like, pro-migratory phenotypes in several tumor models. In colorectal cancer, CD71/TFRC-mediated iron uptake sustains β-catenin/tankyrase activity and tumorigenesis, a pathway intimately tied to EMT and invasion programs [104]. In nasopharyngeal carcinoma TFRC knockdown suppresses PI3K/AKT/mTOR and reduces invasion [82]. CD71 activity is additionally associated with matrix remodeling, including regulation of MMP-2 and MMP-9 in specific contexts, facilitating invasion through basement membranes and stroma [105]. In osteosarcoma tissues, CD71 levels positively correlated with MMP-9, linking CD71 to matrix-degrading capacity [106]. Finally, CD71-mediated iron uptake can indirectly affect migration and angiogenesis by supplying iron to enzymes and pathways that drive motility and vascularization. Iron is a cofactor for prolyl hydroxylases and other dioxygenases that shape HIF signaling, angiogenesis and matrix remodeling—pathways that support migration and invasion [107,108]. Together, TFRC overexpression, pro-migratory/invasive phenotypes and links to EMT/MMP/angiogenic programs position CD71 as a multifunctional driver beyond iron metabolism [30,101]. By intersecting with adhesion and trafficking machinery, CD71 contributes to cytoskeletal dynamics and invasive behavior. This suggests value in pairing CD71-targeted strategies with anti-invasion/EMT or matrix-modulating therapies in tumors with high CD71 expression.

**Figure 2:**
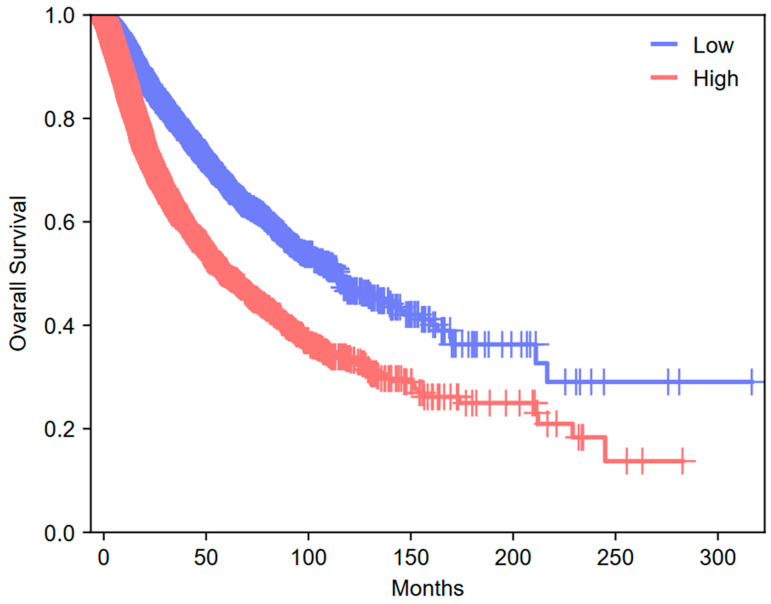
**Kaplan–Meier overall survival by TFRC (CD71) expression (median split).** Kaplan–Meier curves show significantly worse overall survival for the high TFRC group (red) versus low (blue) across the cohort (*n* = 9.290; low *n* = 4.645, high *n* = 4.645). Median OS: 114.0 vs. 58.51 months (low vs. high). Tick marks indicate censored observations; shaded ribbons denote 95% confidence intervals. Log-rank χ^2^ = 261.85, *p* = 6.76 × 10^−59^; univariable Cox HR = 1.86 (95% CI, 1.73–2.01), *p* = 4.14 × 10^−57^. Analysis performed with tools available in GEPIA 3 database [110].

In summary, CD71 may exert widespread influence on tumor growth, survival, and metastasis. These functions, coupled with its overexpression in numerous malignancies, highlight CD71 as a potential therapeutic target. Delineating the molecular mechanisms underlying these non-canonical roles may be critical for developing novel strategies to exploit CD71 in the fight against cancer. This impact is relevant to total data from TCGA consortium [109]. Across 9.290 tumors, we performed analysis on the impact of TFRC expression (median split) on overall survival, as presented in Figure 2. High expression of TFRC was associated with markedly poorer overall survival than low expression (median OS 58.51 vs. 114.0; log-rank χ^2^ = 261.85, *p* = 6.76 × 10^−59^). Consistently, univariable Cox modeling estimated a 1.86-fold higher risk of death for the high-expression group (HR = 1.86, 95% CI 1.73–2.01; *p* = 4.14 × 10^−57^).

## 4. CD71 in the Tumor Microenvironment

The tumor microenvironment (TME) is a highly dynamic and complex ecosystem consisting of cancer cells, stromal cells, immune cells, blood vessels, and extracellular matrix components. These elements interact to create a supportive niche for tumor growth, invasion, and resistance to therapy, which is associated with CD71 biology [111].

### Interaction with Immune Cells

Cancer cells exploit multiple routes to evade immune surveillance, and CD71 is emerging as a modulator of immune responses in the TME [50]. By reshaping iron availability and signaling in immune subsets, CD71 activity can favor an immunosuppressive milieu. Tumors can restrict iron to surrounding immune cells, driving immunosuppressive programming; CD71 is the dominant uptake route for transferrin–iron [60]. One notable effect is on cytotoxic T lymphocytes (CTLs): iron restriction and disrupted CD71-mediated uptake blunt T-cell activation and proliferation, weakening antitumor responses. Blocking CD71 lowers T-cell iron and mTORC1 signaling, impairing effector T-cell programs in vivo [112] and iron deprivation yields non-proliferating T cells despite activation cues [11]. CD71 also influences tumor-associated macrophage (TAM) polarization, skewing programs toward M2-like, tumor-promoting states in iron-restricted contexts [60]. In addition, CD71 expression on regulatory T cells (Tregs) supports their expansion and function in tumors, reinforcing immune suppression. Together, these mechanisms position CD71 as a contributor to immune evasion within the TME [50].

**Figure 3:**
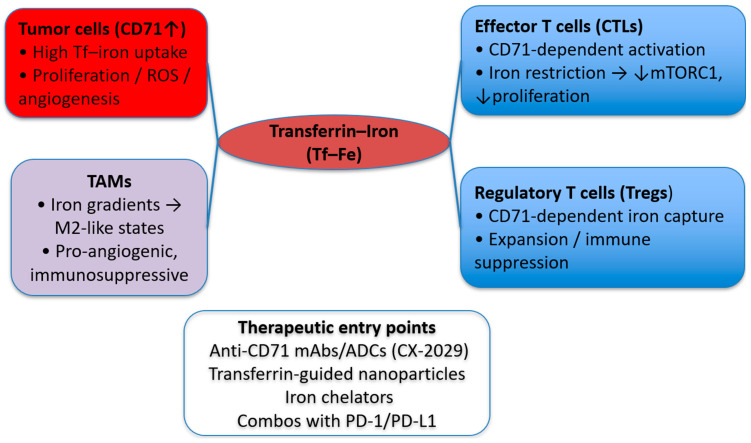
**CD71-centered crosstalk among tumor cells, immune cells, and iron metabolism in the TME.** Tumor cells overexpress CD71, increasing transferrin-bound iron (Tf–Fe) which fuels proliferation, ROS generation, and angiogenesis. Effector T cells require CD71-dependent iron uptake; iron restriction dampens mTORC1 signaling and proliferation, weakening antitumor immunity (arrows indicate increase or decrease). Regulatory T cells (Tregs) capture iron via CD71 to expand and maintain suppressive function, while iron gradients polarize tumor-associated macrophages (TAMs) toward M2-like, pro-angiogenic states. Lines from the Tf–Fe node denote iron supply. The box “Therapeutic entry points” depict putative interventions (anti-CD71 mAbs/ADCs such as CX-2029, transferrin-guided nanocarriers, iron chelators) and potential combinations with PD-1/PD-L1 inhibitors. Abbreviations: Tf, transferrin; Tf–Fe, transferrin–iron; CTL, cytotoxic T lymphocyte; Treg, regulatory T cell; TAM, tumor-associated macrophage; ICI, immune checkpoint inhibitor; ROS, reactive oxygen species; TME, tumor microenvironment.

The cumulative effect of CD71’s interaction with immune cells is the establishment of an immunosuppressive and tumor-promoting environment. Iron-dependent actions of CD71 in the interplay of different cell populations in the TME are depicted in Figure 3.

Several CD71-directed strategies with immunologic relevance have reached the clinic or late preclinical development. The anti-CD71 Probody–drug conjugate CX-2029 has shown first-in-human activity and provided clinical proof-of-targeting for CD71 in solid tumors, while next-generation antibodies and ligand-guided constructs are being explored for immune-stimulatory payload delivery and TME remodeling [68,78,80]. Preclinically, TfR1-targeted immunostimulants and antibody platforms have demonstrated augmentation of antitumor immunity and modulation of the TME, and CD71-dependent iron capture by Tregs has emerged as a mechanistic axis of immune suppression within tumors. As of now, peer-reviewed clinical data on combining CD71-targeted agents with PD-1/PD-L1 blockade are limited. However, early preclinical studies are beginning to test such strategies [78,80]. These findings highlight CD71 as a potential therapeutic target to restore immune surveillance and enhance the efficacy of immunotherapies, such as immune checkpoint inhibitors.

## 5. CD71 and the Crosstalk Between Cancer and Chronic Disease

Beyond tumor-intrinsic effects, CD71-mediated iron trafficking intersects with organ-specific physiology in systems that are exquisitely sensitive to redox balance and mitochondrial demand—most notably the heart, brain, and gastrointestinal tract. Inflammation, hypoxia, and therapy-related stress can remodel the hepcidin–ferroportin axis and shift TFRC/CD71 expression in select cell types (e.g., cardiomyocytes under stress, brain endothelium/progenitors, intestinal epithelium and immune cells), altering iron flux and local oxidative tone. These context-dependent changes help explain how iron dysregulation amplifies dysfunction and injury in these organs while concurrently supporting tumor adaptation. Understanding how CD71 influences both cancer and these chronic conditions is crucial for advancing integrated therapeutic strategies that manage multiple diseases simultaneously [50].

### 5.1. CD71 in Cardiac Disease

Iron is indispensable for cardiovascular health: it supports ATP generation in mitochondria, enables oxygen delivery via hemoglobin, and helps maintain redox balance in cardiomyocytes. Disruption of iron homeostasis, whether via deficiency or overload, may lead to a significant cardiac dysfunction. While the transferrin receptor/CD71 is central to cellular iron uptake and regulation in many tissues, its role in cardiomyocytes may be particularly consequential in pathological states.

Iron deficiency (ID), even in the absence of anemia, is highly prevalent in patients with cardiovascular disease, especially heart failure [113,114,115]. By limiting mitochondrial electron transport and reducing available iron for enzymes, ID may impair myocardial energetics, diminish contractile reserve, and exacerbate symptoms of heart failure [114]. In the setting of chronic disease or cancer, systemic inflammation often increases hepcidin levels, further restricting iron bioavailability and worsening functional iron deficiency [115]. Intravenous iron repletion has shown benefits in selected heart failure patients, improving symptoms, exercise capacity, and reducing hospitalizations [116].

Conversely, excessive iron uptake by cardiomyocytes can drive cardiomyopathy via oxidative stress, mitochondrial injury, disruption of mitochondrial dynamics, and apoptosis [117,118,119]. In models of iron overload cardiomyopathy, iron deposition within myocardium leads to diastolic dysfunction, arrhythmias, and, eventually, dilated cardiomyopathy [120]. While direct evidence linking CD71 overexpression in the heart to exacerbated iron accumulation is still limited, it is plausible that enhanced CD71-mediated uptake under pathological circumstances (e.g., chronic inflammation or supplementation) could worsen myocardial iron loading and drive damage.

In patients with cancer, anemia of chronic disease (ACD) is common. Inflammatory cytokines elevate hepcidin levels, sequester iron in macrophages, and limit iron export, culminating in functional iron restriction. The resulting iron deficiency imposes additional strain on the heart, particularly when cancer therapies (e.g., anthracyclines, targeted agents) themselves have cardiotoxic potential. In this context, iron dysregulation becomes a two-fold threat. It undermines systemic oxygen delivery and predisposes the myocardium to injury [119]. Therefore, in cancer care it is critical to monitor iron status not only for optimizing erythropoiesis and tumor response but also for safeguarding cardiac function, especially in patients receiving potentially cardiotoxic treatments. Further studies are needed to provide a direct link between CD71, Cancer progression and cardiovascular disease.

### 5.2. CD71 in Neuronal Disease

Iron is essential for brain function, supporting neurotransmitter synthesis, myelination, and neuronal energy metabolism. Iron imbalance is closely tied to cognitive decline and neurodegeneration.

Dysregulated iron homeostasis promotes oxidative stress and lipid peroxidation leading to ferroptosis, an iron-dependent type of cell-death. The above mechanism may be implicated in the pathogenesis of common neurodegenerative disease, such as Alzheimer’s disease (AD), Parkinson’s disease (PD), amyotrophic lateral sclerosis (ALS), and related disorders [121,122,123].

CD71/transferrin receptor-1 is the principal iron-uptake route in the CNS. It is highly expressed on brain endothelial cells at the blood–brain barrier, which is a major gateway for iron and for CD71-mediated transport. It is present on proliferating neural progenitors, while being downregulated on mature neurons, while the physiological expression patterns become perturbed in disease [124,125,126].

In AD models, cortical CD71 protein is increased alongside HIF-1 pathway activation, consistent with a stress-related iron uptake. Human and experimental studies link brain iron overload to Aβ/tau pathology and oxidative damage. PD cohorts show abnormalities in iron handling with changes involving ferritin/TfR pathways. Collectively, CD71 dysregulation in disease-relevant brain cells (endothelium, glia, progenitors and, under stress, some neurons) can amplify iron influx, ROS deposition, lipid peroxidation and cell death programs that induce neuroinflammation and neurodegeneration [127,128]. The existence of CD71 in plasma neural-derived exosomes suggest that they may act as potential biomarkers with implications for neurological disease pathology [129].

### 5.3. CD71 in Gastrointestinal Disease

CD71 is a principal iron-uptake route in the Gastrointestinal (GI) tract and is frequently dysregulated across several gastrointestinal malignancies. In gastric cancer, CD71 is overexpressed in cancerous tissues compared to matched non-cancerous ones, based on immunohistochemistry analysis, and associates with significantly worse overall survival [130]. Colorectal cancer (CRC) exhibits disease-relevant CD71 activity, including a non-canonical nuclear CD71 translocation that enhances nucleotide-excision–repair and tracks with aggressiveness and metastasis [131]. In hepatocellular carcinoma (HCC), CD71 promotes cancer progression via mTOR signaling and correlates with poorer overall survival and disease-free survival [132]. Pancreatic ductal adenocarcinoma (PDAC) shows elevated CD71 in tumors and also on circulating neutrophils, both linked to worse prognosis [133,134]. Collectively, CD71 upregulation supports iron dependency, redox stress signaling, and invasive behavior across GI tumors.

In inflammatory bowel disease (IBD), mucosal CD71 expression rises on both basolateral and apical enterocyte membranes and is shaped by inflammation-driven HIF-1α signaling, which in turn can induce CD71 and alter iron handling. In that manner a positive feedback loop links inflammation to iron homeostasis. Specifically, inflammatory signaling stabilizes HIF-1α and activates the IRP/IRE response, which upregulates TFRC/CD71 and increases epithelial iron uptake. The rise in labile iron and ROS further activates NF-κB and stabilizes HIF-1α, amplifying cytokine production and sustaining TFRC induction. These adaptations may help explain functional iron deficiency and variable responses to oral iron in active disease [135,136].

Therapeutically, CD71 is being associated with targeted cytotoxicity in GI cancers. A The CX-2029 anti-CD71 Probody drug conjugate has shown first-in-human activity with a strategy that limits off-tumor uptake, and is under continued clinical evaluation [78], following a succesfull preclinical evaluation in preclinical non-human models [137]. The antibody is conjugated to monomethyl auristatin E (MMAE) and engineered to be protease-activatable in the tumor microenvironment, thereby restricting exposure to normal tissues and achieving specificity to cancer tissue. Complementary approaches like H-ferritin/CD71-targeted nanocarriers demonstrate GI-tumor selectivity in preclinical models [138].

### 5.4. Summary on CD71 in Chronic Disease

Collectively, current evidence supports CD71 might have implications beyond cancer biology and being associated with comorbidities in cardiological, neurological and gastrointestinal systems. Clinically, this notion suggests for integrated monitoring of iron status and organ function alongside oncologic care, and for precision interventions applied with attention to off-tumor effects and patient heterogeneity. Future trials may evaluate CD71 expression and iron-inflammation biomarkers, incorporate long-term organ surveillance, and test combinations such as immunotherapy or ferroptosis modulators that optimize outcomes while minimizing toxicity.

From a clinical viewpoint, modulating CD71 may affect both cancer and comorbid chronic diseases that depend on iron balance. Anti-CD71/iron-restrictive approaches could theoretically intensify iron-limited states warranting comorbidity-aware selection and laboratory monitoring (hemoglobin, ferritin, transferrin saturation). Conversely, TfR1-based shuttles may facilitate CNS drug delivery in neuro-oncology but require dosing strategies that minimize peripheral off-target effects. These considerations support integrated trial designs that take into account the comorbidities and a multidisciplinary management that aligns anti-tumor efficacy with organ safety. An overview of current CD71 therapeutic approaches is presented in Table 1.

## 6. Methods

Statistical Methods for Survival Analyses. Overall survival (OS) analyses for TFRC (CD71) used TCGA RNA-seq expression and clinical outcome data accessed via GEPIA 3 [110] (Pan-Cancer module; default TCGA cohort aggregation). Expression units were *log2(TPM + 1)* as provided by GEPIA. Cohort: *n* = 9.290 primary tumors with OS and expression available. Patients were split by the cohort median TFRC expression (high vs. low). Survival curves were estimated by Kaplan–Meier; differences were assessed by two-sided log-rank test with χ^2^ reported. Effect sizes were estimated by univariable Cox proportional hazards models, reporting HR and 95% CI. Time scale: months; censoring as per TCGA clinical files. Parameters: GEPIA 3 defaults; Pan-Cancer; OS endpoint; median cutoff. GEPIA 3 was assessed at 14 October 2025.

## 7. Conclusions

Recent advancements in understanding CD71’s functions have illuminated its potential roles as a diagnostic and/or prognostic biomarker, as well as potential therapeutic target. By refining our understanding of CD71’s involvement in cancer and chronic disease, and by developing tailored therapeutic strategies that address both tumor growth and the health of other organ systems, we can improve patient outcomes and advance integrated disease management.

The evidence provided in this report supports that CD71 is a multifunctional protein that plays significant roles in cancer biology. Traditionally recognized for its involvement in iron metabolism, CD71’s functions extend beyond the regulation of cellular iron homeostasis. Its contributions to key cellular processes such as signal transduction, apoptosis, autophagy, and metastasis underline its critical involvement in driving cancer progression. CD71 also has an impact on cellular proliferation, survival, and the ability of tumor cells to invade and spread. Moreover, CD71 is significant in the broader tumor microenvironment, where it modulates immune responses and contributes to chronic inflammation. CD71-mediated mechanisms, particularly its effect on immune cells, can support tumor immune evasion, creating an immunosuppressive environment that enhances tumor survival and growth. Additionally, CD71’s role in the regulation of iron homeostasis intersects with chronic diseases, notably in the cardiovascular and neurological systems, complicating cancer treatment.

Collectively, current data support CD71 as a candidate diagnostic and/or prognostic biomarker and a tractable therapeutic target, provided that strategies account for tumor context and organ health. These activities also underscore both opportunities and safety considerations for translation, given the intersection of CD71-driven biology with chronic disease mechanisms. Recent advancements in understanding CD71’s functions have illuminated its potential roles as a diagnostic and/or prognostic biomarker, as well as potential therapeutic target. By refining our understanding of CD71’s involvement in cancer and chronic disease, and by developing tailored therapeutic strategies that address both tumor growth and the health of other organ systems, we can improve patient outcomes and advance integrated disease management. Looking ahead, future studies should define the therapeutic window for CD71-targeted agents and develop engineering/dosing strategies that minimize off-tumor uptake, establish standardized assays and clinically useful cutoffs for TFRC/CD71 as a biomarker in multivariable, tumor-specific cohorts, map non-canonical signaling partners and crosstalk (HIF, MAPK/ERK, PI3K/AKT) to identify druggable nodes, test rational combinations with immunotherapy and targeted agents using mechanism-based pharmacodynamic endpoints, and integrate comorbidity-aware translation by assessing how CD71 modulation impacts iron balance and organ function in patients with concomitant chronic diseases.

## Figures and Tables

**Table 1:** CD71-targeted therapeutic approaches.

Therapeutic Approach	Example	Mechanism	Development/Status	Key Pros/Cons	Refs
Monoclonal antibodies/ADCs/Probody–drug conjugates	Anti-CD71 mAbs; CX-2029 (anti-CD71 Probody–MMAE); TFRC gene-silencing approaches	Block CD71 or deliver cytotoxins via CD71-mediated internalization; may reduce iron uptake or kill CD71-high cells	CX-2029: first-in-human Phase I completed; nonclinical efficacy/safety reported	High tumor uptake, Probody masks reduce off-tumor effects; risks of off-tumor binding in proliferative tissues	[68,78,80,137]
Transferrin–drug conjugates (Tf–DCs)	Tf–doxorubicin conjugates; TfR-targeted peptide–doxorubicin conjugates	Use endogenous transferrin/CD71 pathway to ferry chemotherapeutics into tumor cells	preclinical and early translational efforts	Leverages physiologic iron pathway; potential competition with endogenous transferrin, heterogeneity of CD71 expression	[69,70,71,77]
Iron chelators/ferroptosis-linked strategies	Deferasirox; deferoxamine	Deprive cells of bioavailable iron and/or tilt cells toward ferroptotic vulnerability	Repurposing and combination strategies explored	Conceptually tumor-agnostic; systemic iron depletion risks (anemia, off-target effects)	[72,73]
TfR-targeted nanoparticles/liposomes	Tf- liposomes; TfR ligands; H-ferritin nanocarriers	CD71-mediated endocytosis concentrates payloads in tumors and/or across barriers	Preclinical/early clinical feasibility reported for several platforms	Selective uptake; manufacturing, off-tumor uptake	[74,75,76,138]
Peptide/aptamer binders to CD71	Short TfR-targeting peptides	Compact ligands for targeting and internalization via CD71	Preclinical reports with in vitro efficacy	Small ligands; shorter half-life or weaker affinity than mAbs	[77]
CD71 shuttles for brain delivery (BBB crossing)	Bispecific anti-TfR1 ‘shuttles’; TfR-guided carriers	Exploit endothelial CD71 at the BBB	Neurodegeneration drug delivery	CNS access; risk of peripheral sink/anemia	[125]

## Data Availability

No new data were created or analyzed in this study.

## References

[1] Bray F., Laversanne M., Sung H., Ferlay J., Siegel R.L., Soerjomataram I., Jemal A. (2024). Global cancer statistics 2022: GLOBOCAN estimates of incidence and mortality worldwide for 36 cancers in 185 countries. CA A Cancer J. Clin..

[2] Force L.M., Kocarnik J.M., May M.L., Bhangdia K., Crist A., Penberthy L., Pritchett N., Acheson A., Deitesfeld L., A B. (2025). The global, regional, and national burden of cancer, 1990–2023, with forecasts to 2050: A systematic analysis for the Global Burden of Disease Study 2023. Lancet.

[3] Lyons V.J., Helms A., Pappas D. (2019). The effect of protein expression on cancer cell capture using the Human Transferrin Receptor (CD71) as an affinity ligand. Anal. Chim. Acta.

[4] Gennery A.R., Rezaei N. (2022). Chapter 5—Autoimmunity in combined immunodeficiency. Translational Autoimmunity.

[5] Rodriguez R., Schreiber S.L., Conrad M. (2022). Persister cancer cells: Iron addiction and vulnerability to ferroptosis. Mol. Cell.

[6] Basuli D., Tesfay L., Deng Z., Paul B., Yamamoto Y., Ning G., Xian W., McKeon F., Lynch M., Crum C.P. (2017). Iron addiction: A novel therapeutic target in ovarian cancer. Oncogene.

[7] Gammella E., Buratti P., Cairo G., Recalcati S. (2017). The transferrin receptor: The cellular iron gate. Met. Integr. Biometal Sci..

[8] Kawabata H. (2019). Transferrin and transferrin receptors update. Free. Radic. Biol. Med..

[9] Wang S., He X., Wu Q., Jiang L., Chen L., Yu Y., Zhang P., Huang X., Wang J., Ju Z. (2020). Transferrin receptor 1-mediated iron uptake plays an essential role in hematopoiesis. Haematologica.

[10] Ned R.e.M., Swat W., Andrews N.C. (2003). Transferrin receptor 1 is differentially required in lymphocyte development. Blood.

[11] Berg V., Modak M., Brell J., Puck A., Künig S., Jutz S., Steinberger P., Zlabinger G.J., Stöckl J. (2020). Iron Deprivation in Human T Cells Induces Nonproliferating Accessory Helper Cells. ImmunoHorizons.

[12] Aba Ü., Maslak İ.C., İpşir C., Pehlivan D., Warnock N.I., Tumes D.J., Cildir G., Erman B. (2024). A Novel Homozygous Germline Mutation in Transferrin Receptor 1 (TfR1) Leads to Combined Immunodeficiency and Provides New Insights into Iron-Immunity Axis. J. Clin. Immunol..

[13] von Haehling S. (2025). Iron deficiency in heart failure: Epidemiology, diagnostic criteria and treatment modalities. ESC Heart Fail..

[14] Jujić A., Molvin J., Holm Isholth H., Dieden A., Korduner J., Zaghi A., Nezami Z., Bergmann A., Schomburg L., Magnusson M. (2024). Association between low selenoprotein P concentrations and anaemia in hospitalized heart failure patients. ESC Heart Fail..

[15] Kozłowska B., Sochanowicz B., Kraj L., Palusińska M., Kołsut P., Szymański Ł., Lewicki S., Śmigielski W., Kruszewski M., Leszek P. (2022). Expression of Iron Metabolism Proteins in Patients with Chronic Heart Failure. J. Clin. Med..

[16] Zhan Y., Zhou Y., Zhang C., Zhai Z., Yang Y., Liu X. (2025). Transferrin receptor knockdown attenuates atrial fibrillation by inhibiting cardiomyocyte ferroptosis and atrial fibrosis. Exp. Anim..

[17] Tian S., Wang B., Ding Y., Zhang Y., Yu P., Chang Y.-Z., Gao G. (2024). The role of iron transporters and regulators in Alzheimer’s disease and Parkinson’s disease: Pathophysiological insights and therapeutic prospects. Biomed. Pharmacother..

[18] Zeng W., Cai J., Zhang L., Peng Q. (2024). Iron Deposition in Parkinson’s Disease: A Mini-Review. Cell. Mol. Neurobiol..

[19] Pfeifhofer-Obermair C., Tymoszuk P., Petzer V., Weiss G., Nairz M. (2018). Iron in the Tumor Microenvironment—Connecting the Dots. Front. Oncol..

[20] Wang Y., Yu L., Ding J., Chen Y. (2018). Iron Metabolism in Cancer. Int. J. Mol. Sci..

[21] Guo Q., Qian C., Wang X., Qian Z.-M. (2025). Transferrin receptors. Exp. Mol. Med..

[22] Aisen P. (2004). Transferrin receptor 1. Int. J. Biochem. Cell Biol..

[23] MacKenzie E.L., Iwasaki K., Tsuji Y. (2008). Intracellular iron transport and storage: From molecular mechanisms to health implications. Antioxid. Redox Signal..

[24] Eckenroth B.E., Steere A.N., Chasteen N.D., Everse S.J., Mason A.B. (2011). How the binding of human transferrin primes the transferrin receptor potentiating iron release at endosomal pH. Proc. Natl. Acad. Sci. USA.

[25] West A.R., Oates P.S. (2008). Mechanisms of heme iron absorption: Current questions and controversies. World J. Gastroenterol..

[26] Faubert B., Solmonson A., DeBerardinis R.J. (2020). Metabolic reprogramming and cancer progression. Science.

[27] Ward P.S., Thompson C.B. (2012). Metabolic reprogramming: A cancer hallmark even warburg did not anticipate. Cancer Cell.

[28] Manz D.H., Blanchette N.L., Paul B.T., Torti F.M., Torti S.V. (2016). Iron and cancer: Recent insights. Ann. N. Y. Acad. Sci..

[29] Hsu M.Y., Mina E., Roetto A., Porporato P.E. (2020). Iron: An Essential Element of Cancer Metabolism. Cells.

[30] Shen Y., Li X., Dong D., Zhang B., Xue Y., Shang P. (2018). Transferrin receptor 1 in cancer: A new sight for cancer therapy. Am. J. Cancer Res..

[31] Habashy H.O., Powe D.G., Staka C.M., Rakha E.A., Ball G., Green A.R., Aleskandarany M., Paish E.C., Douglas Macmillan R., Nicholson R.I. (2010). Transferrin receptor (CD71) is a marker of poor prognosis in breast cancer and can predict response to tamoxifen. Breast Cancer Res. Treat..

[32] Wu Y., Xu J., Chen J., Zou M., Rusidanmu A., Yang R. (2018). Blocking transferrin receptor inhibits the growth of lung adenocarcinoma cells in vitro. Thorac. Cancer.

[33] Adachi M., Kai K., Yamaji K., Ide T., Noshiro H., Kawaguchi A., Aishima S. (2019). Transferrin receptor 1 overexpression is associated with tumour de-differentiation and acts as a potential prognostic indicator of hepatocellular carcinoma. Histopathology.

[34] Keer H.N., Kozlowski J.M., Tsai Y.C., Lee C., McEwan R.N., Grayhack J.T. (1990). Elevated transferrin receptor content in human prostate cancer cell lines assessed in vitro and in vivo. J. Urol..

[35] Zhuang C., Li X., Yang L., Ma X., Shen Y., Huang C., Pan T., Cui J., Ni B., Wang M. (2023). Overexpressed transferrin receptor implied poor prognosis and relapse in gastrointestinal stromal tumors. Front. Oncol..

[36] Semenza G.L. (2001). Hypoxia-Inducible Factor 1: Control of Oxygen Homeostasis in Health and Disease. Pediatr. Res..

[37] Lok C.N., Ponka P. (1999). Identification of a Hypoxia Response Element in the Transferrin Receptor Gene*. J. Biol. Chem..

[38] Bianchi L., Tacchini L., Cairo G. (1999). HIF-1-mediated activation of transferrin receptor gene transcription by iron chelation. Nucleic Acids Res..

[39] Tacchini L., Bianchi L., Bernelli-Zazzera A., Cairo G. (1999). Transferrin receptor induction by hypoxia. HIF-1-mediated transcriptional activation and cell-specific post-transcriptional regulation. J. Biol. Chem..

[40] O’Donnell K.A., Yu D., Zeller K.I., Kim J.W., Racke F., Thomas-Tikhonenko A., Dang C.V. (2006). Activation of transferrin receptor 1 by c-Myc enhances cellular proliferation and tumorigenesis. Mol. Cell. Biol..

[41] Yang W.S., Stockwell B.R. (2008). Synthetic Lethal Screening Identifies Compounds Activating Iron-Dependent, Nonapoptotic Cell Death in Oncogenic-RAS-Harboring Cancer Cells. Chem. Biol..

[42] Chen C., Liu P., Duan X., Cheng M., Xu L.X. (2019). Deferoxamine-induced high expression of TfR1 and DMT1 enhanced iron uptake in triple-negative breast cancer cells by activating IL-6/PI3K/AKT pathway. OncoTargets Ther..

[43] Iommarini L., Porcelli A.M., Gasparre G., Kurelac I. (2017). Non-Canonical Mechanisms Regulating Hypoxia-Inducible Factor 1 Alpha in Cancer. Front. Oncol..

[44] Malekan M., Ebrahimzadeh M.A., Sheida F. (2021). The role of Hypoxia-Inducible Factor-1alpha and its signaling in melanoma. Biomed. Pharmacother..

[45] Li Y., Sun X.X., Qian D.Z., Dai M.S. (2020). Molecular Crosstalk Between MYC and HIF in Cancer. Front. Cell Dev. Biol..

[46] Doe M.R., Ascano J.M., Kaur M., Cole M.D. (2012). Myc posttranscriptionally induces HIF1 protein and target gene expression in normal and cancer cells. Cancer Res..

[47] Perez G., Barber G.P., Benet-Pages A., Casper J., Clawson H., Diekhans M., Fischer C., Gonzalez J.N., Hinrichs A.S., Lee C.M. (2025). The UCSC genome browser database: 2025 update. Nucleic Acids Res..

[48] Moore J.E., Pratt H.E., Fan K., Phalke N., Fisher J., Elhajjajy S.I., Andrews G., Gao M., Shedd N., Fu Y. (2024). An expanded Registry of candidate cis-Regulatory Elements for studying transcriptional regulation. bioRxiv.

[49] Rauluseviciute I., Riudavets-Puig R., Blanc-Mathieu R., Castro-Mondragon J.A., Ferenc K., Kumar V., Lemma R.B., Lucas J., Chèneby J., Baranasic D. (2024). JASPAR 2024: 20th anniversary of the open-access database of transcription factor binding profiles. Nucleic Acids Res..

[50] Bu X., Wang L. (2025). Iron metabolism and the tumor microenvironment: A new perspective on cancer intervention and therapy (Review). Int. J. Mol. Med..

[51] Ying J.F., Lu Z.B., Fu L.Q., Tong Y., Wang Z., Li W.F., Mou X.Z. (2021). The role of iron homeostasis and iron-mediated ROS in cancer. Am. J. Cancer Res..

[52] Zhao Z. (2023). Hydroxyl radical generations form the physiologically relevant Fenton-like reactions. Free. Radic. Biol. Med..

[53] Perillo B., Di Donato M., Pezone A., Di Zazzo E., Giovannelli P., Galasso G., Castoria G., Migliaccio A. (2020). ROS in cancer therapy: The bright side of the moon. Exp. Mol. Med..

[54] Nakamura H., Takada K. (2021). Reactive oxygen species in cancer: Current findings and future directions. Cancer Sci..

[55] Brandl N., Seitz R., Sendtner N., Müller M., Gülow K. (2025). Living on the Edge: ROS Homeostasis in Cancer Cells and Its Potential as a Therapeutic Target. Antioxidants.

[56] Tiwari R., Mondal Y., Bharadwaj K., Mahajan M., Mondal S., Sarkar A. (2025). Reactive Oxygen Species (ROS) and Their Profound Influence on Regulating Diverse Aspects of Cancer: A Concise Review. Drug Dev. Res..

[57] Jian J., Yang Q., Dai J., Eckard J., Axelrod D., Smith J., Huang X. (2011). Effects of iron deficiency and iron overload on angiogenesis and oxidative stress-a potential dual role for iron in breast cancer. Free. Radic. Biol. Med..

[58] He H., Qiao Y., Zhou Q., Wang Z., Chen X., Liu D., Yin D., He M. (2019). Iron Overload Damages the Endothelial Mitochondria via the ROS/ADMA/DDAHII/eNOS/NO Pathway. Oxidative Med. Cell. Longev..

[59] Mu W., Zhou Z., Shao L., Wang Q., Feng W., Tang Y., He Y., Wang Y. (2023). Advances in the relationship between ferroptosis and epithelial–mesenchymal transition in cancer. Front. Oncol..

[60] Sun J.L., Zhang N.P., Xu R.C., Zhang G.C., Liu Z.Y., Abuduwaili W., Wang F., Yu X.N., Shi X., Song G.Q. (2021). Tumor cell-imposed iron restriction drives immunosuppressive polarization of tumor-associated macrophages. J. Transl. Med..

[61] Liang W., Ferrara N. (2021). Iron Metabolism in the Tumor Microenvironment: Contributions of Innate Immune Cells. Front. Immunol..

[62] Jung M., Weigert A., Mertens C., Rehwald C., Brüne B. (2017). Iron Handling in Tumor-Associated Macrophages-Is There a New Role for Lipocalin-2?. Front. Immunol..

[63] Ni S., Yuan Y., Kuang Y., Li X. (2022). Iron Metabolism and Immune Regulation. Front. Immunol..

[64] Teh M.R., Frost J.N., Armitage A.E., Drakesmith H. (2021). Analysis of Iron and Iron-Interacting Protein Dynamics During T-Cell Activation. Front. Immunol..

[65] Sacco A., Battaglia A.M., Botta C., Aversa I., Mancuso S., Costanzo F., Biamonte F. (2021). Iron Metabolism in the Tumor Microenvironment-Implications for Anti-Cancer Immune Response. Cells.

[66] Zhang Y.-Y., Han Y., Li W.-N., Xu R.-H., Ju H.-Q. (2024). Tumor iron homeostasis and immune regulation. Trends Pharmacol. Sci..

[67] Pacella I., Pinzon Grimaldos A., Rossi A., Tucci G., Zagaglioni M., Potenza E., Pinna V., Rotella I., Cammarata I., Cancila V. (2024). Iron capture through CD71 drives perinatal and tumor-associated Treg expansion. JCI Insight.

[68] Candelaria P.V., Leoh L.S., Penichet M.L., Daniels-Wells T.R. (2021). Antibodies Targeting the Transferrin Receptor 1 (TfR1) as Direct Anti-cancer Agents. Front. Immunol..

[69] Daniels T.R., Bernabeu E., Rodríguez J.A., Patel S., Kozman M., Chiappetta D.A., Holler E., Ljubimova J.Y., Helguera G., Penichet M.L. (2012). The transferrin receptor and the targeted delivery of therapeutic agents against cancer. Biochim. Biophys. Acta (BBA)—Gen. Subj..

[70] Łubgan D., Jóźwiak Z., Grabenbauer G.G., Distel L.V. (2009). Doxorubicin-transferrin conjugate selectively overcomes multidrug resistance in leukaemia cells. Cell. Mol. Biol. Lett..

[71] Wigner P., Zielinski K., Labieniec-Watala M., Marczak A., Szwed M. (2021). Doxorubicin–transferrin conjugate alters mitochondrial homeostasis and energy metabolism in human breast cancer cells. Sci. Rep..

[72] Ibrahim O., O’Sullivan J. (2020). Iron chelators in cancer therapy. Biometals.

[73] Lui G.Y.L., Obeidy P., Ford S.J., Tselepis C., Sharp D.M., Jansson P.J., Kalinowski D.S., Kovacevic Z., Lovejoy D.B., Richardson D.R. (2013). The Iron Chelator, Deferasirox, as a Novel Strategy for Cancer Treatment: Oral Activity Against Human Lung Tumor Xenografts and Molecular Mechanism of Action. Mol. Pharmacol..

[74] Daniels T.R., Delgado T., Rodriguez J.A., Helguera G., Penichet M.L. (2006). The transferrin receptor part I: Biology and targeting with cytotoxic antibodies for the treatment of cancer. Clin. Immunol..

[75] Mojarad-Jabali S., Mahdinloo S., Farshbaf M., Sarfraz M., Fatahi Y., Atyabi F., Valizadeh H. (2022). Transferrin receptor-mediated liposomal drug delivery: Recent trends in targeted therapy of cancer. Expert Opin. Drug Deliv..

[76] Wang J., Tian S., Petros R.A., Napier M.E., DeSimone J.M. (2010). The Complex Role of Multivalency in Nanoparticles Targeting the Transferrin Receptor for Cancer Therapies. J. Am. Chem. Soc..

[77] Yu J., Mao X., Yang X., Zhao G., Li S. (2024). New Transferrin Receptor-Targeted Peptide-Doxorubicin Conjugates: Synthesis and In Vitro Antitumor Activity. Molecules.

[78] Johnson M., El-Khoueiry A., Hafez N., Lakhani N., Mamdani H., Rodon J., Sanborn R.E., Garcia-Corbacho J., Boni V., Stroh M. (2021). Phase I, First-in-Human Study of the Probody Therapeutic CX-2029 in Adults with Advanced Solid Tumor Malignancies. Clin. Cancer Res. Off. J. Am. Assoc. Cancer Res..

[79] Chen A.C., Donovan A., Ned-Sykes R., Andrews N.C. (2015). Noncanonical role of transferrin receptor 1 is essential for intestinal homeostasis. Proc. Natl. Acad. Sci. USA.

[80] Campisi A., Bonfanti R., Raciti G., Bonaventura G., Legnani L., Magro G., Pennisi M., Russo G., Chiacchio M.A., Pappalardo F. (2020). Gene Silencing of Transferrin-1 Receptor as a Potential Therapeutic Target for Human Follicular and Anaplastic Thyroid Cancer. Mol. Ther. Oncolytics.

[81] Sánchez M.F., Tampé R. (2023). Ligand-independent receptor clustering modulates transmembrane signaling: A new paradigm. Trends Biochem. Sci..

[82] Feng G., Arima Y., Midorikawa K., Kobayashi H., Oikawa S., Zhao W., Zhang Z., Takeuchi K., Murata M. (2023). Knockdown of TFRC suppressed the progression of nasopharyngeal carcinoma by downregulating the PI3K/Akt/mTOR pathway. Cancer Cell Int..

[83] Chan K.T., Choi M.Y., Lai K.K.Y., Tan W., Tung L.N., Lam H.Y., Tong D.K.H., Lee N.P., Law S. (2014). Overexpression of transferrin receptor CD71 and its tumorigenic properties in esophageal squamous cell carcinoma. Oncol. Rep..

[84] Chen J., Fu Y., Li Y., Weng S., Wang H., He J., Dong C. (2025). Transferrin receptor 1 (TfR1) functions as an entry receptor for scale drop disease virus to invade the host cell via clathrin-mediated endocytosis. J. Virol..

[85] Vartholomatos E., Mantziou S., Alexiou G.A., Lazari D., Sioka C., Kyritsis A., Markopoulos G.S. (2022). An NF-κB- and Therapy-Related Regulatory Network in Glioma: A Potential Mechanism of Action for Natural Antiglioma Agents. Biomedicines.

[86] Shenoy G., Connor J.R. (2023). A closer look at the role of iron in glioblastoma. Neuro-Oncology.

[87] Xu G., Wen X., Hong Y., Du H., Zhang X., Song J., Yin Y., Huang H., Shen G. (2011). An anti-transferrin receptor antibody enhanced the growth inhibitory effects of chemotherapeutic drugs on human glioma cells. Int. Immunopharmacol..

[88] Kawak P., Sawaftah N.M.A., Pitt W.G., Husseini G.A. (2023). Transferrin-Targeted Liposomes in Glioblastoma Therapy: A Review. Int. J. Mol. Sci..

[89] Lazari D., Alexiou G.A., Markopoulos G.S., Vartholomatos E., Hodaj E., Chousidis I., Leonardos I., Galani V., Kyritsis A.P. (2017). N-(p-coumaroyl) serotonin inhibits glioblastoma cells growth through triggering S-phase arrest and apoptosis. J. Neuro-Oncol..

[90] Vartholomatos E., Alexiou G.A., Markopoulos G.S., Lazari D., Tsiftsoglou O., Chousidis I., Leonardos I., Kyritsis A.P. (2020). Deglucohellebrin: A Potent Agent for Glioblastoma Treatment. Anti-Cancer Agents Med. Chem..

[91] Jian J., Yang Q., Huang X. (2011). Src regulates Tyr(20) phosphorylation of transferrin receptor-1 and potentiates breast cancer cell survival. J. Biol. Chem..

[92] Wang J., An W., Pang Z., Zhao M., Xu A., Zhao J. (2025). The TFRC as a prognostic biomarker and potential therapeutic target in cervical cancer: A preliminary study. Front. Oncol..

[93] Vogler M., Braun Y., Smith V.M., Westhoff M.A., Pereira R.S., Pieper N.M., Anders M., Callens M., Vervliet T., Abbas M. (2025). The BCL2 family: From apoptosis mechanisms to new advances in targeted therapy. Signal Transduct. Target. Ther..

[94] Kasibhatla S., Jessen K.A., Maliartchouk S., Wang J.Y., English N.M., Drewe J., Qiu L., Archer S.P., Ponce A.E., Sirisoma N. (2005). A role for transferrin receptor in triggering apoptosis when targeted with gambogic acid. Proc. Natl. Acad. Sci. USA.

[95] Delbridge A.R.D., Strasser A. (2015). The BCL-2 protein family, BH3-mimetics and cancer therapy. Cell Death Differ..

[96] Puri C., Park S.J., Wrobel L., Rubinsztein D.C. (2025). Transferrin receptor controls both autophagosome formation and closure via phosphatidylinositol 3-phosphate synthesis. Dev. Cell.

[97] Moharir S.C., Sirohi K., Swarup G. (2023). Regulation of transferrin receptor trafficking by optineurin and its disease-associated mutants. Prog. Mol. Biol. Transl. Sci..

[98] Mancias J.D., Wang X., Gygi S.P., Harper J.W., Kimmelman A.C. (2014). Quantitative proteomics identifies NCOA4 as the cargo receptor mediating ferritinophagy. Nature.

[99] White E., Lattime E.C., Guo J.Y. (2021). Autophagy Regulates Stress Responses, Metabolism, and Anticancer Immunity. Trends Cancer.

[100] Russell R.C., Guan K.L. (2022). The multifaceted role of autophagy in cancer. EMBO J..

[101] Ren G., Zhou J., Su Y., Yang Q., Li J. (2025). TFRC promotes the proliferation, migration, and invasion of osteosarcoma cells by increasing the intracellular iron content and RRM2 expression. Front. Oncol..

[102] Riggs K.A., Hasan N., Humphrey D., Raleigh C., Nevitt C., Corbin D., Hu C. (2012). Regulation of integrin endocytic recycling and chemotactic cell migration by syntaxin 6 and VAMP3 interaction. J. Cell Sci..

[103] Huang Y., Hong W., Wei X. (2022). The molecular mechanisms and therapeutic strategies of EMT in tumor progression and metastasis. J. Hematol. Oncol..

[104] Kim H., Villareal L.B., Liu Z., Haneef M., Falcon D.M., Martin D.R., Lee H.J., Dame M.K., Attili D., Chen Y. (2023). Transferrin Receptor-Mediated Iron Uptake Promotes Colon Tumorigenesis. Adv. Sci..

[105] Jiang H., Li H. (2021). Prognostic values of tumoral MMP2 and MMP9 overexpression in breast cancer: A systematic review and meta-analysis. BMC Cancer.

[106] Bai C., Ma X., Wang X., Chen X. (2022). Correlation between pathological features and protein expressions of TfR1, VEGF and MMP-9 in patients with osteosarcoma. Am. J. Transl. Res..

[107] Torti S.V., Torti F.M. (2020). Iron and Cancer: 2020 Vision. Cancer Res..

[108] Dürig J., Calcagni M., Buschmann J. (2023). Transition metals in angiogenesis—A narrative review. Mater. Today Bio.

[109] Weinstein J.N., Collisson E.A., Mills G.B., Shaw K.R., Ozenberger B.A., Ellrott K., Shmulevich I., Sander C., Stuart J.M. (2013). The cancer genome atlas pan-cancer analysis project. Nat. Genet..

[110] Kang Y.-J., Pan L., Liu Y., Rong Z., Liu J., Liu F. (2025). GEPIA3: Enhanced drug sensitivity and interaction network analysis for cancer research. Nucleic Acids Res..

[111] Arneth B. (2019). Tumor Microenvironment. Medicina.

[112] Voss K., Sewell A.E., Krystofiak E.S., Gibson-Corley K.N., Young A.C., Basham J.H., Sugiura A., Arner E.N., Beavers W.N., Kunkle D.E. (2023). Elevated transferrin receptor impairs T cell metabolism and function in systemic lupus erythematosus. Sci. Immunol..

[113] Savarese G., von Haehling S., Butler J., Cleland J.G.F., Ponikowski P., Anker S.D. (2023). Iron deficiency and cardiovascular disease. Eur. Heart J..

[114] Othon-Martínez D., Fernandez-Betances O.A., Málaga-Espinoza B.X., Torres-Perez M.E., Cobos E., Gutierrez-Martinez C. (2024). Iron and cardiovascular health: A review. J. Investig. Med. Off. Publ. Am. Fed. Clin. Res..

[115] Lakhal-Littleton S., Cleland J.G.F. (2024). Iron deficiency and supplementation in heart failure. Nat. Rev. Cardiol..

[116] Cheema B., Chokshi A., Orimoloye O., Ardehali H. (2024). Intravenous Iron Repletion for Patients with Heart Failure and Iron Deficiency. JACC.

[117] Kumfu S., Chattipakorn S.C., Chattipakorn N. (2022). Iron overload cardiomyopathy: Using the latest evidence to inform future applications. Exp. Biol. Med..

[118] Rhee J.W., Yi H., Thomas D., Lam C.K., Belbachir N., Tian L., Qin X., Malisa J., Lau E., Paik D.T. (2020). Modeling Secondary Iron Overload Cardiomyopathy with Human Induced Pluripotent Stem Cell-Derived Cardiomyocytes. Cell Rep..

[119] Li S., Zhang X. (2021). Iron in Cardiovascular Disease: Challenges and Potentials. Front. Cardiovasc. Med..

[120] Agrawal A., El Dahdah J., Haroun E., Arockiam A.D., Safdar A., Sorathia S., Dong T., Griffin B., Wang T.K.M. (2025). A Contemporary Review of Clinical Manifestations, Evaluation, and Management of Cardiac Complications of Iron Overload. Hearts.

[121] Gao Q., Zhou Y., Chen Y., Hu W., Jin W., Zhou C., Yuan H., Li J., Lin Z., Lin W. (2025). Role of iron in brain development, aging, and neurodegenerative diseases. Ann. Med..

[122] Houldsworth A. (2024). Role of oxidative stress in neurodegenerative disorders: A review of reactive oxygen species and prevention by antioxidants. Brain Commun..

[123] Fei Y., Ding Y. (2024). The role of ferroptosis in neurodegenerative diseases. Front. Cell. Neurosci..

[124] Thomsen M.S., Johnsen K.B., Kucharz K., Lauritzen M., Moos T. (2022). Blood-Brain Barrier Transport of Transferrin Receptor-Targeted Nanoparticles. Pharmaceutics.

[125] Shen X., Li H., Zhang B., Li Y., Zhu Z. (2025). Targeting Transferrin Receptor 1 for Enhancing Drug Delivery Through the Blood–Brain Barrier for Alzheimer’s Disease. Int. J. Mol. Sci..

[126] Menon V., Thomas R., Elgueta C., Horl M., Osborn T., Hallett P.J., Bartos M., Isacson O., Pruszak J. (2019). Comprehensive Cell Surface Antigen Analysis Identifies Transferrin Receptor Protein-1 (CD71) as a Negative Selection Marker for Human Neuronal Cells. Stem Cells.

[127] Petralla S., Saveleva L., Kanninen K.M., Oster J.S., Panayotova M., Fricker G., Puris E. (2024). Increased Expression of Transferrin Receptor 1 in the Brain Cortex of 5xFAD Mouse Model of Alzheimer’s Disease Is Associated with Activation of HIF-1 Signaling Pathway. Mol. Neurobiol..

[128] Levi S., Ripamonti M., Moro A.S., Cozzi A. (2024). Iron imbalance in neurodegeneration. Mol. Psychiatry.

[129] Chen Z.-t., Pan C.-z., Ruan X.-l., Lei L.-p., Lin S.-m., Wang Y.-z., Zhao Z.-H. (2023). Evaluation of ferritin and TfR level in plasma neural-derived exosomes as potential markers of Parkinson’s disease. Front. Aging Neurosci..

[130] Cao J., Hu C., Xu J., Han J., Zhang R., Cao M., Yuan L., Xu Z. (2022). Aberrant Expression TFR1/CD71 in Gastric Cancer Identifies a Novel Potential Prognostic Marker and Therapeutic Target. Evid.-Based Complement. Altern. Med. Ecam.

[131] Hou Y., Tang G., Wang Q., Zhou M., Xu R., Chen X., Shi G., Wang Z., Yan X., Zhuang J. (2025). Transferrin receptor 1 nuclear translocation facilitates tumor progression via p53-mediated chromatin interactions and genome-wide alterations. Signal Transduct. Target. Ther..

[132] Wang F., Xu W.Q., Zhang W.Q., Xu R.C., Sun J.L., Zhang G.C., Liu Z.Y., Qi Z.R., Dong L., Weng S.Q. (2024). Transferrin receptor 1 promotes hepatocellular carcinoma progression and metastasis by activating the mTOR signaling pathway. Hepatol. Int..

[133] Yang C., Li J., Guo Y., Gan D., Zhang C., Wang R., Hua L., Zhu L., Ma P., Shi J. (2022). Role of TFRC as a Novel Prognostic Biomarker and in Immunotherapy for Pancreatic Carcinoma. Front. Mol. Biosci..

[134] Hansen F.J., Mittelstädt A., Clausen F.-N., Knoedler S., Knoedler L., Klöckner S., Kuchenreuther I., Mazurie J., Arnold L.-S., Anthuber A. (2024). CD71 expressing circulating neutrophils serve as a novel prognostic biomarker for metastatic spread and reduced outcome in pancreatic ductal adenocarcinoma patients. Sci. Rep..

[135] Harel E., Rubinstein A., Nissan A., Khazanov E., Nadler Milbauer M., Barenholz Y., Tirosh B. (2011). Enhanced Transferrin Receptor Expression by Proinflammatory Cytokines in Enterocytes as a Means for Local Delivery of Drugs to Inflamed Gut Mucosa. PLoS ONE.

[136] Fagundes R.R., Bourgonje A.R., Hu S., Barbieri R., Jansen B.H., Sinnema N., Blokzijl T., Taylor C.T., Weersma R.K., Faber K.N. (2022). HIF1α-Dependent Induction of TFRC by a Combination of Intestinal Inflammation and Systemic Iron Deficiency in Inflammatory Bowel Disease. Front. Physiol..

[137] Singh S., Serwer L., DuPage A., Elkins K., Chauhan N., Ravn M., Buchanan F., Wang L., Krimm M., Wong K. (2022). Nonclinical Efficacy and Safety of CX-2029, an Anti-CD71 Probody-Drug Conjugate. Mol. Cancer Ther..

[138] Cheng X., Fan K., Wang L., Ying X., Sanders A.J., Guo T., Xing X., Zhou M., Du H., Hu Y. (2020). TfR1 binding with H-ferritin nanocarrier achieves prognostic diagnosis and enhances the therapeutic efficacy in clinical gastric cancer. Cell Death Dis..

